# Brain-Restricted Inhibition of IL-6 Trans-Signaling Mildly Affects Metabolic Consequences of Maternal Obesity in Male Offspring

**DOI:** 10.3390/nu13113735

**Published:** 2021-10-23

**Authors:** Saida Breuer, Philipp Kasper, Christina Vohlen, Ruth Janoschek, Thorben Hoffmann, Sarah Appel, Elena Müller-Limberger, Andrea Mesaros, Stefan Rose-John, Christoph Garbers, Stefan Müller, Jan-Wilm Lackmann, Esther Mahabir, Jörg Dötsch, Eva Hucklenbruch-Rother, Inga Bae-Gartz

**Affiliations:** 1 Department of Pediatrics and Adolescent Medicine, Faculty of Medicine and University Hospital Cologne, University of Cologne, D-50937 Cologne, Germany; saida.breuer@uk-koeln.de (S.B.); christina.vohlen@uk-koeln.de (C.V.); ruth.janoschek@uk-koeln.de (R.J.); thorben.hoffmann@uk-koeln.de (T.H.); sarah.appel@uk-koeln.de (S.A.); elena.mueller-limberger@uk-koeln.de (E.M.-L.); joerg.doetsch@uk-koeln.de (J.D.); eva.rother@uni-koeln.de (E.H.-R.); 2 Clinic for Gastroenterology and Hepatology, Faculty of Medicine and University Hospital Cologne, University of Cologne, D-50937 Cologne, Germany; philipp.kasper@uk-koeln.de; 3 Department of Phenotyping, Max-Planck Institute for Biology of Aging, University of Cologne, D-50931 Cologne, Germany; andrea.mesaros@age.mpg.de; 4 Department for Biochemistry, Christian-Albrechts-University zu Kiel, D-24098 Kiel, Germany; rosejohn@biochem.uni-kiel.de; 5 Department of Pathology, Medical Faculty, Otto-von-Guericke-University Magdeburg, D-39120 Magdeburg, Germany; christoph.garbers@med.ovgu.de; 6 Center for Molecular Medicine (CMMC), Proteomics Facility, University of Cologne, D-50931 Cologne, Germany; stefan.mueller@uni-koeln.de; 7 Cologne Excellence Cluster on Cellular Stress Responses in Aging-Associated Diseases (CECAD), University of Cologne, D-50931 Cologne, Germany; jan-wilm.lackmann@uni-koeln.de; 8 Comparative Medicine, Center for Molecular Medicine Cologne (CMMC), Faculty of Medicine and University Hospital Cologne, D-50937 Cologne, Germany; esther.mahabir-brenner@uni-koeln.de

**Keywords:** perinatal programming, maternal nutrition, newborn nutrition, IL-6-trans-signaling, hypothalamic dysfunction, prevention, western-style-diet, GFAP (glial fibrillary acidic protein), sgp130 (soluble gp130), proteomics

## Abstract

Maternal obesity greatly affects next generations, elevating obesity risk in the offspring through perinatal programming and flawed maternal and newborn nutrition. The exact underlying mechanisms are poorly understood. Interleukin-6 (IL-6) mediates its effects through a membrane-bound receptor or by trans-signaling (tS), which can be inhibited by the soluble form of the co-receptor gp130 (sgp130). As IL-6 tS mediates western-style diet (WSD) effects via chronic low-grade inflammation (LGI) and LGI is an important mediator in brain–adipose tissue communication, this study aims at determining the effects of maternal obesity in a transgenic mouse model of brain-restricted IL-6tS inhibition (^GFAPsgp130^) on offspring’s short- and long-term body composition and epigonadal white adipose tissue (egWAT) metabolism. Female wild type (WT) or transgenic mice were fed either standard diet (SD) or WSD pregestationally, during gestation, and lactation. Male offspring received SD from postnatal day (P)21 to P56 and were metabolically challenged with WSD from P56 to P120. At P21, offspring from WT and transgenic dams that were fed WSD displayed increased body weight and egWAT mass, while glucose tolerance testing showed the strongest impairment in ^GFAPsgp130^WSD offspring. Simultaneously, egWAT proteome reveals a characteristic egWAT expression pattern in offspring as a result of maternal conditions. IL-6tS inhibition in transgenic mice was in tendency associated with lower body weight in dams on SD and their respective offspring but blunted by the WSD. In conclusion, maternal nutrition affects offspring’s body weight and egWAT metabolism predominantly independent of IL-6tS inhibition, emphasizing the importance of maternal and newborn nutrition for long-term offspring health.

## 1. Introduction

Maternal obesity is a substantial problem as it does not only affect the mother’s wellbeing, but it also increases the risk for birth complications and health problems in their children and, thus, generations to come [1,2]. Poor maternal nutritional conditions during fetal development and early life induce both short-term and long-term adverse metabolic effects in the offspring associated with an increased risk of obesity and type 2 diabetes (T2D). Therefore, newborn nutrition is crucially influenced by maternal nutritional status starting from pregnancy and lactation in modulating fetal, neonatal, and infant growth. This concept is described as perinatal programming [3,4]. In order to understand the underlying mechanism of perinatal programming, shedding light on the organs involved in and affected by maternal obesity during critical periods is vital. Because adipose tissue is directly affected by obesity through expansion [5], this tissue is an important player to be considered. An excess of visceral or epigonadal (eg) white adipose tissue (WAT) is linked to systemic low-grade inflammation (LGI), and WAT has been discovered as a very active, endocrine organ part-taking in the changes in metabolism seen in obese individuals [6]. LGI in the obese state is linked to inflammatory markers, amongst which interleukin-6 (IL-6) and monocyte chemoattractant protein 1 (MCP-1) have received much attention to date [7]. The hypothalamus is the brain’s center of hunger and satiety, is known to integrate WAT-derived signaling peptides in order to regulate the body’s energy homeostasis [8]. The “fat-brain-axis” is of particular relevance in the context of better understanding the exact mechanisms by which maternal obesity might affect the offspring’s hypothalamic development and function and, thus, long-term energy homeostasis. Understanding the effects of WAT-linked LGI [9,10,11] on the hypothalamus might help develop therapeutic and preventive strategies with hopefully greater compliance than dietary changes [12].

Specifically, deciphering the role of the LGI marker IL-6 in obesity has been emphasized over the past decade [13,14]. IL-6 is a cytokine that is involved in physiologic and pathologic states, including neurodegeneration, trauma, infection and immune cell proliferation and differentiation [13,15]. IL-6 activates cells via a classical signaling complex existing in the transmembrane IL-6 α receptor (IL-6Rα) and the signal-transducing β-subunit glycoprotein 130 (gp130) [16]. This central IL-6 signaling is responsible for the transmission of mainly anti-inflammatory effects of IL-6. Although gp130 is ubiquitously expressed in the central nervous system, membrane-bound IL-6Rα expression is specific to hepatocytes, neutrophils, monocytes, and macrophages. Cells that do not express IL-6R respond to IL-6 in the presence of agonistic soluble IL-6R (sIL-6R). The sIL6R-IL6 complex can bind to gp130, which also results in intracellular signaling. This process is called IL-6 trans-signaling (IL-6tS) and has been shown to be responsible for the transmission of the pro-inflammatory effects of IL-6 [17,18]. IL-6tS can be selectively blocked by a soluble form of gp130 (sgp130) without affecting IL-6 signaling via the membrane-bound IL-6R [16,19].

Knowledge of the neurological impact of IL-6 has come from a transgenic model (named GFAP-IL6) with astrocyte production of IL-6 [14] and from a transgenic model (named ^GFAPspg130^ here) with astrocyte production of spg130 [14]. While GFAP-IL6 mice presented localized neuroinflammation and neurodegenerative disorders, ^GFAPsgp130^mice could alleviate many of the detrimental effects of IL-6 [14].

Particularly during critical time periods, such as the perinatal period, IL-6 can have severe impacts on the neuronal plasticity and brain. Previous data have demonstrated that mouse offspring of obese dams displays increased hypothalamic IL-6 signaling, predominately mediated by IL-6tS [20,21]. However, there is a lack of data on the effects of a brain-restricted IL-6tS transgenic mouse model on whole-body metabolism and on peripheral organs such as white adipose tissue as an important metabolic organ.

In general, data on a potential “hypothalamus-fat axis” are still scarce. Recently, Wang and colleagues demonstrated that activation of hypothalamic rat-insulin-promotor-Cre neurons promotes beiging of WAT via the sympathetic nervous system [22]. Further, Campbell et al. could show that IL-6tS is responsible for CNS pathologies using the same animal model as in this study [14]. In detail, the authors were able to show that blocking of IL-6tS in the brain alleviates many of the detrimental effects of IL-6, such as dysbalanced angiogenesis, blood–brain barrier leakage, gliosis, impaired hippocampal neurogenesis and degenerative changes in the cerebellum [14]. Some of these pathologies, especially gliosis and altered neurogenesis, are known underlying causes of hypothalamic dysfunction in obese animals as well as in offspring of obese dams [8,23,24,25,26,27]. Thus, brain-specific IL-6tS seems to be a promising target for the prevention or treatment of hypothalamic dysfunction and consequential metabolic disturbances in the offspring of obese dams. In addition, impairment of the hypothalamus–pituitary–adrenal axis by diet-induced obesity is linked to hypercortisolism which in turn affects the glucocorticoid receptors in adipose tissue. This shows a direct effect of hypothalamic changes on egWAT metabolism [28]. The influential role of IL-6 as a cytokine that controls body weight by signals from the adipose tissue is summarized by Mohamed-Ali et al., indicating that IL-6 is able to induce metabolic alterations via the hypothalamus [29]. The regulatory role of IL-6 in adipose tissue inflammation was also established [30]. As hypothalamic inflammation occurs before the body weight gain, which in turn elevates egWAT inflammation [31], it is reasonable that IL-6 induced inflammation in the hypothalamus, at least in part, impacts adipose tissue growth and adipose tissue inflammation.

To date, there are only limited data on the effects of hypothalamic IL-6tS on metabolic consequences in peripheral organs such as WAT. Therefore, we set out to characterize the effect of brain-restricted IL-6tS inhibition on the offspring’s energy metabolism and egWAT function. We subjected WT and transgenic mice with a brain-restricted inhibition of IL-6tS to maternal obesity pregestationally during pregnancy and lactation. We hypothesized that brain-restricted inhibition of IL-6tS might protect the offspring from IL-6tS-mediated long-term adverse metabolic consequences following maternal obesity.

## 2. Materials and Methods

### 2.1. Animal Model

Our study was carried out by the Department of Pediatrics of the University Hospital of Cologne. The study was approved by the appropriate governmental authority (Institutional protocol number of the animal welfare application: AZ 81.02.04.2017.A442, Landesamt für Natur, Umwelt und Verbraucherschutz Nordrhein-Westfalen, Germany). All animal procedures were performed in accordance with the German Animal Welfare Law. Animal care and use were performed by qualified individuals supervised by a veterinarian. The manuscript complied with the Animals in Research: Reporting In Vivo Experiments (ARRIVE) guidelines [32]. The two diets that were used in this study were the standard diet (SD) and the western-style diet (WSD) (For detailed information, see Supplementary Table S1). WT mice (C57BL/6N) were purchased from Charles River Laboratories, Germany. Transgenic glial fibrillary acidic protein (GFAP) soluble glycoprotein 130 (sgp130) fused with the crystallizable fragment (FC) of IgG1 to increase binding capacity, shortened ^GFAPsgp130^, mice were obtained from Prof. Rose-John, Department for Biochemistry, Christian-Albrechts University zu Kiel. ^GFAPsgp130^mice (C57BL/6N) were generated and characterized as previously described [14]. Briefly, sgp130 was expressed under the control of the GFAP promoter expressed in astrocytes. The sgp130 protein specifically inhibited IL-6 trans-signaling but not IL-6 classic signaling [16]. Campbell and colleagues demonstrated that ^GFAPsgp130^mice produced sgp130 in the brain and that the produced sgp130 by astrocytes was able to bind IL-6/sIL-6R complexes [14].

Mice were bred locally at a designated animal unit of the University Hospital of Cologne (Cologne, Germany) and were housed individually and maintained at 22 °C on a 12-h light–12-h dark cycle (6:00 a.m. to 6:00 p.m.).

After the transport to our animal unit, ^GFAPsgp130^mice were kept on an SD to acclimatize for 6 months and become used to the hygienic situation in the animal facility. As wild type (WT) and transgenic mice both had a C57BL/6N background, ^GFAPsgp130^mice were not backcrossed again to wild types purchased from Charles River.

**Figure 1:**
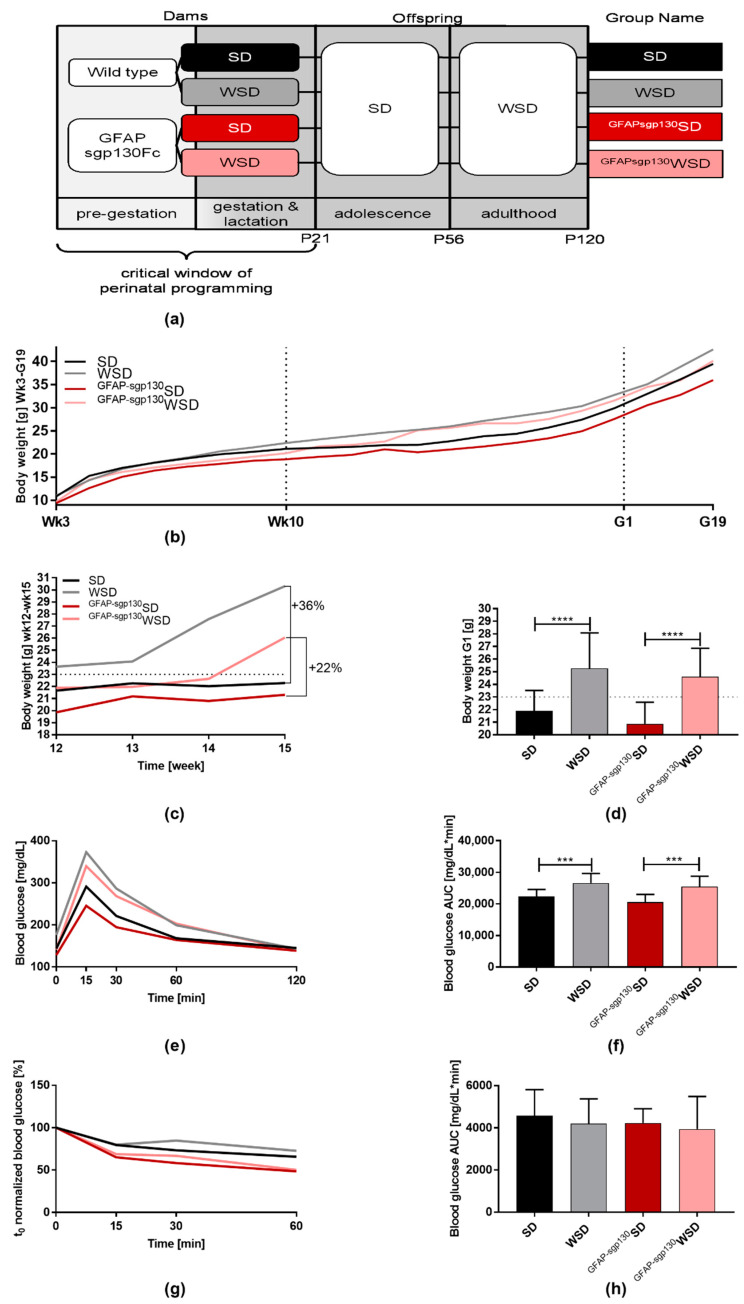
Metabolic characterization of dams. Data are presented as mean or mean + SD; *** *p* < 0.001; **** *p* < 0.0001. (**a**) Experimental set-up and feeding protocol; standard diet (SD), western-style diet (WSD); (**b**) weight progression from week 3 (wk3) to gestational day 19 (G19). *n* = 33–58/group; (**c**) weight progression from week (wk) 12 to wk15. *n* = 8–32/group; (**d**) body weight at gestational day 1 (G1). *n* = 33–58/group; (**e**) intraperitoneal glucose tolerance test (ipGTT) and (**f**) area under the curve (AUC) at week 10. *n* = 10/group; (**g**) intraperitoneal insulin tolerance test (ipITT); (**h**) area under the curve (AUC) at week 10. *n* = 10/group.

Three-week-old female WT and ^GFAPsgp130^ mice were fed an SD or WSD for 10–12 weeks, at preconception, and during gestation and lactation, resulting in four groups: WT mice on the standard chow diet, labeled SD; WT mice on WSD, labeled WSD; transgenic mice on SD, labeled ^GFAPsgp130^SD; and transgenic mice on WSD, labeled ^GFAPsgp130^WSD. Offspring was labeled in correspondence to their mothers. Dams were weighed weekly until mating, SD dams weighed below 23 g, and WSD dams weighed above 23 g. Dams were weighed every other day during gestation, starting on the day of mating, gestational day 1 (G1). The birth weight of offspring was collected on P1 within 24 h, and weight progression in the first 21 days of the pups was collected every other day and weekly after P21. On P3, litter size was adjusted to six for each litter. The study model is shown in Figure 1a. After P21, pups were weaned and distributed to same-sex cages depending on the experimental starting point to ensure an equal time spent with comrades. We ensured that litter mates were randomly ascribed to different time points and experiments to exclude potential litter effects. From P56 to P120, all offspring were subjected to a WSD for 10 weeks. For the duration of the whole study, all mice had free access to the experimental diets and water, with the exception of food fasting periods before tests described below.

### 2.2. Analytical Procedures

Offspring were sacrificed via CO_2_-inhalation at P21, P56, and P120 or via cervical dislocation at P21 for micro computed-tomography (µCT) analysis. All dams were sacrificed via cervical dislocation after lactation. Dams and offspring were weighed at sacrifice. Blood was extracted by cardiac puncture, kept at room temperature (RT) for 30 min, and then centrifuged for 10 min at 3000× *g* and 4 °C for serum collection. Serum was kept at 80 °C until analyzed. The egWAT was weighed and immediately frozen in liquid nitrogen and kept at −80 °C. A maximum of one pup per dam was analyzed per sub-experiment to exclude litter-dependent bias.

### 2.3. Food Intake (FI) and Food Preference (FP) Test

In order to measure FI in offspring, cages were provided with food racks. FI was determined over a period of five consecutive days in dams during weeks eight to 10 and in offspring from P60 to P65. For FP testing in offspring, two food racks were placed into the cage simultaneously, containing either SD or WSD. Mice from the same group were kept together in groups of three during the experiment to exclude a decline in food intake due to loneliness. The mean consumption was calculated for each mouse.

### 2.4. Intraperitoneal Glucose Tolerance Test (ipGTT) and Intraperitoneal Insulin Tolerance Test (ipITT)

The ipGTT and ipITT were executed after a 6 h fasting period (7:00 a.m.–1:00 p.m.). 2 mg glucose/kg body weight or 0.75 mU insulin/gram body weight was injected intraperitoneally (i.p.). Blood glucose was measured after 0, 15, 30, and 60 min of injection for the ipGTT and ipITT and additionally after 120 min for the ipGTT.

### 2.5. Proteomics Analysis

For protein isolation, egWAT was isolated as previously described [25]. Briefly, frozen tissue was homogenized with a tissue grinder in lysis buffer described previously [33]. Protein was precipitated with acetone overnight (ON) at −20 °C and further digested with lysl-endopeptidase (Lys-C) and trypsin. Detailed preparation of probes was described previously [34]. Following the stage-tip procedure, as described previously [35], samples were stored at −20 °C prior to analysis. Analyses took place on an Orbitrap Exploris 480 (Thermo Scientific, Waltham, MA, USA) mass spectrometer equipped with a FAIMSpro differential ion mobility device coupled to an Easy 1200 nano LC (all Thermo Scientific). Samples were loaded onto an in-house pulled and packed 30 cm analytical column (inner diameter 75 µm, filled with 2.7 µm Poroshell EC120 C18, Agilent). Peptides were chromatographically separated at a constant flow rate of 300 nL/min over the following gradient running eluent A (0.1% formic acid) against eluent B (0.1% formic acid in 80% acetonitrile). Starting at 4% B, up to 30% B in 74 min, up to 55% B in 8 min, up to 95% B in 2 min, followed by a 6 min column wash with 95% solvent B and re-equilibration. The FAIMS pro was operated at −47 V compensation voltage and 99.5 °C (inner) and 85 °C (outer) electrode temperatures. Identical HPLC settings were used for library generation and sample runs.

For spectrum library generation by gas phase fractionation, aliquots from each sample were pooled, and the pool was used for spectrum library generation by narrow window data-independent acquisition (DIA) of six 100 m/z gas phase fractions (GPF) covering the range from 400 m/z to 1000 m/z [36]. The Orbitrap was operated in DIA mode. MS1 scans of the respective 100 m/z gas phase fraction were acquired at 60 k resolution. Maximum injection time was set to 60 ms and the AGC target to 100%. MS2 scans of the corresponding 100 m/z regions were acquired in 24 × 4 m/z staggered windows resulting in 48 nominal 2 m/z windows after demultiplexing. MS2 settings were 30 k resolution, 60 ms maximum injection time and an AGC target of 1000% and 30% HCD collision energy. All scans were stored as centroid.

The DIA of samples was performed by acquiring MS1 scans from 390 m/z to 1010 m/z at 60 k resolution. Maximum injection time was set to 60 ms and the AGC target to 100%. MS2 scans ranged from 300 m/z to 1800 m/z and were acquired at 15 k resolution with a maximum injection time of 22 msec, an AGC target of 1000%, and 30% HCD collision energy. DIA scans covered the precursor range from 400 to 1000 m/z and were acquired in 50 × 12 m/z staggered windows resulting in 100 nominal 6 m/z windows after demultiplexing. All scans were stored as a centroid. For proteomic data processing, thermo raw files were demultiplexed and transformed to mzML files using the msconvert module in Proteowizard. MzML files were converted to *.dia file format in DIA-NN 1.7.18. For spectral library generation, a mouse canonical Swissprot FASTA file was converted to a Prosit upload file with the convert tool in encyclopedia 0.9.0 [37] using default settings: Trypsin, up to 1 missed cleavage, range 396 m/z–1004 m/z, charge states 2+ and 3+, default charge state 3 and NCE 33. The .csv file was uploaded to the Prosit webserver and converted to a spectrum library in generic text format (Gessulat 2019). The resulting library (16,998 protein isoforms, 21,693 protein groups, and 1,404,872 precursors) was searched in DIA-NN 1.7.18 [38] with the 6 GPF runs to generate a project-specific library (8961 protein isoforms, 9487 protein groups, and 72,634 precursors). The applied settings were as follows: output is filtered at 0.01 FDR, N-terminal methionine excision enabled, the maximum number of missed cleavages set to 1, min peptide length set to 7, max peptide length set to 30, min precursor m/z set to 400, max precursor m/z set to 1000, cysteine carbamidomethylation enabled as a fixed modification.

Thirty-six sample files were searched with DIA-NN 1.7.18 against the project library. In addition to the settings used for library generation, and the results were filtered on a protein group q-value and global q-value of 0.01. RT-dependent normalization and relaxed protein inference option were used.

### 2.6. Quantitative PCR

The egWAT RNA was isolated using TRI-Reagent^®^ (Sigma-Aldrich) according to the manufacturer’s guidelines. Tecan spectrophotometer (Tecan, Nano Quant infinite M200 Pro) was used for measurement of concentration and quality of RNA. The 7500 Realtime PCR system (Applied Biosystem, Foster City, CA, USA) was used for the measurement of quantitative changes in mRNA expression as described before [25]. Three housekeeping genes were tested (*18S*, *Gapdh*, ß-*Actin* (*bAct*)) to normalize genes of interest. For primer pairs and taqman probes, please refer to Supplementary Table S2.

### 2.7. ELISA Analysis

Serum was analyzed with the ELISA kits Milliplex MAP Mouse Adipokine Magnetic Bead Panel-Endocrine Multiplex Assay (#MADKMAG-71K), MOUSE SOLUBLE CYTOKINE RECEPTOR MAGNETIC BEAD PANEL (#MSCRMAG-42K), and Milliplex MAP Mouse Aging Magnetic Bead Panel 1 (#MAGE1MAG-25K) by Merck Millipore following the manufacture’s guidelines for the following markers: IL-6, sIL-6R, insulin, leptin, MCP-1 and FGF-21. The values of the samples were included if they are within the manufacturer’s minimum detectable concentration (IL-6: 2.3 ± 6.3 pg/mL, sIL-6R: 24.1 pg/mL, insulin: 13.0 ± 27.7 pg/mL, leptin: 4.2 ± 8.2 pg/mL, MCP-1: 4.9 ± 11.9 pg/mL, and FGF-21: 3.5 ± 5.6 pg/mL).

### 2.8. µ CTanalysis

For exact measurements and visualization of total and visceral fat depots, mice were sacrificed by cervical dislocation, cadavers were frozen and handed to the Max Plank Institute for Biology of Aging, Cologne, Germany, and analyzed as previously described [27]. Briefly, X-rays were performed over 360° with a rotation step of 0.6° to ensure total capture of fat depots. Based on tissue density, images were segmented for total volume and fat volume, subdivided into total and visceral fat.

### 2.9. Statistical Analysis

Statistics were conducted with GraphPad version 7. Grubb’s outlier test was performed on data for each group individually. Data were tested for equal distribution using the D’Agostino-Pearson omnibus normality test.

A two-way ANOVA was carried out. If significance was reached, a post-hoc Bonferroni test was calculated to analyze differences between the two groups. Following two-group comparisons were regarded: SD vs. WSD, ^GFAPsgp130^SD vs. ^GFAPsgp130^WSD, SD vs. ^GFAPsgp130^SD, and WSD vs. ^GFAPsgp130^WSD. Comparisons were chosen beforehand based on meaningfulness for our hypothesis. Further two-group comparisons were not regarded as functionally relevant. Data were shown as mean+ standard deviation. The ipITT progression results are shown normalized to blood glucose levels at 0 min. qPCR calculations were completed with the ΔΔCT method and shown as a fold change to the control group SD in all figures. Statistical significance was defined as *p* < 0.05.

Proteomic data analysis was performed with Perseus version 1.6.15 [39]. The generic matrix was uploaded, and data were lg2(x) transformed. Only proteins with five valid values in at least one group were accepted for further analysis. Missing values were replaced by using Impute LCMD and q (MinDet) = 0.01, and data from this matrix are shown in a PCA with components 2 (13.5%) and 5 (5.9%) to show clustering dependent on less pronounced components. A two-way ANOVA was analyzed. Results were Bonferroni-corrected. All proteins with significantly different expressions due to diet were filtered and analyzed with reactome.org version 77. Proteins that differed by maternal diet or genotype were separately regarded with reactome.org version 77. Significantly differed pathways were derived from Voronoi visualization, with the selection “all non-human identifiers are converted to their human equivalents” [40].

## 3. Results

### 3.1. Brain-Restricted Inhibition of IL-6tS Mildly Attenuates WSD Impact on Dams

In order to assess maternal weight gain and development of glucose intolerance, all dams underwent weekly weighing from weaning until mating and every other day from mating until birth (Figure 1a). Additionally, food intake was determined at 8 to 10 weeks of age, and at the age of 10 weeks, ipGTT and ipITT were performed. Figure 1b,c is included as a descriptive overview of overall body weight in dams. On average, transgenic dams needed longer to reach obesity on WSD. At week 12, ^GFAPsgp130^mice revealed a lower body weight than WT mice, comparing the respective diets. ^GFAPsgp130^WSD dams only gained 22% of body weight compared to a 36% weight gain in WSD dams (Figure 1c). When entering pregnancy, dams on WSD displayed significantly higher body weight than those on SD in both transgenic and WT animals. Diet and genotype showed significant influence as determined by two-way ANOVA. Here, there is no longer a significant difference in body weight comparing ^GFAPsgp130^dams and WT dams on respective diets at G1 (Figure 1d). Quantification of daily food intake revealed a higher caloric intake of both WSD groups compared to the respective SD groups, but no statistically significant difference between WSD and ^GFAPsgp130^WSD dams. During ipGTT, WSD and ^GFAPsgp130^WSD dams reached higher blood glucose levels than SD and ^GFAPsgp130^SD dams, respectively, as indicated by significantly increased area under the curve (AUC) values, with significant overall influence by diet (Figure 1e,f). However, there were no significant differences in insulin sensitivity assessed by ipITT detectable (Figure 1g,h). Mean± standard deviation for line graphs is provided in Supplementary Table S3.

Taken together, both WSD groups of dams were significantly heavier and less glucose tolerant entering pregnancy.

### 3.2. Maternal Diet Shows Effects on Offspring’s Body Weight and egWAT Percentage with Only Partial Effects of Brain-Restricted Inhibition of IL-6tS

**Figure 2:**
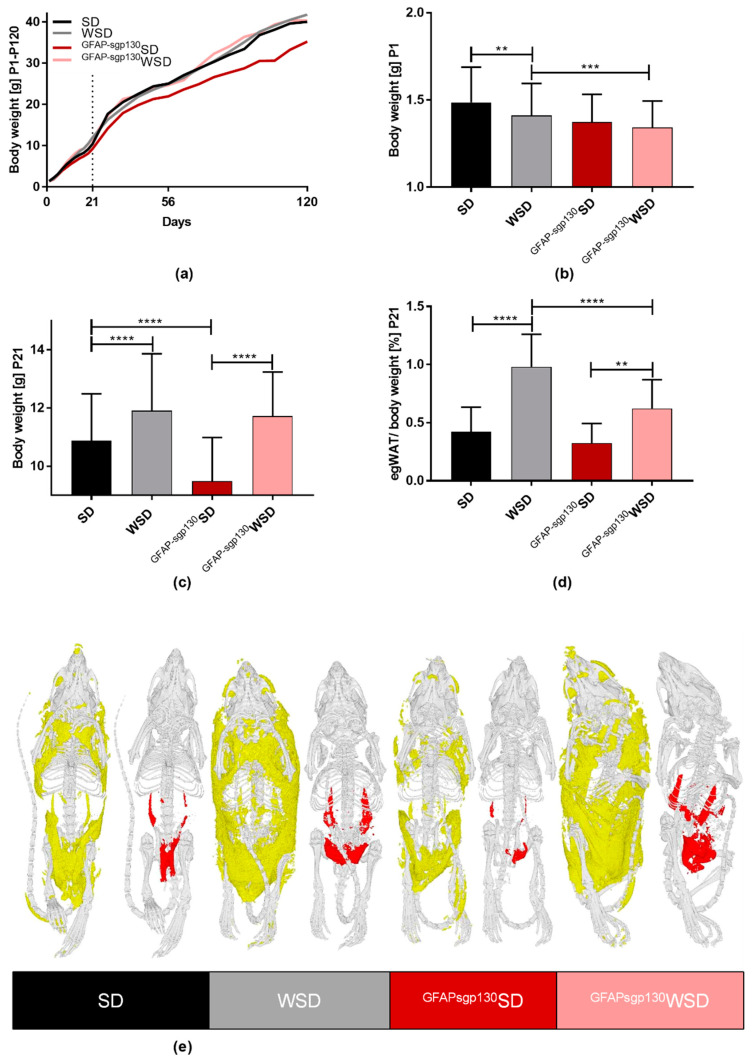
Phenotyping of male offspring at P1 and P21. Data are presented as mean + SD; ** *p* < 0.01; *** *p* < 0.001; **** *p* < 0.0001. (**a**) weight progression between birth and P120. *n* = 64–130/group; (**b**) birth weight. *n* = 64–130/group; (**c**) body weight at P21. *n* = 57–129/group; (**d**) epigonadal white adipose tissue (egWAT) percentage at P21. *n* = 24–54/group; (**e**) representative µCT measurements for the determination of total (yellow) and visceral (red) fat distribution in offspring at P21, left to right: SD, WSD, ^GFAPsgp130^SD, ^GFAPsgp130^WSD. *n* = 1/group shown as a representative of 5/group collected.

In order to determine the impact of maternal diet and metabolism on offspring phenotype, offspring’s body weight was monitored every second day during the first three weeks of life and weekly from P21 to P120 (Figure 2a; for mean ± standard variation, see Supplementary Table S4). At P1, both maternal diet and genotype showed a significant effect. WSD offspring was significantly lighter than SD offspring (Figure 2b). This effect was not found in ^GFAPsgp130^WSD offspring compared to ^GFAPsgp130^SD offspring. However, ^GFAPsgp130^WSD offspring was significantly lighter than WSD offspring at P1 (Figure 2b). At P21, both WSD and ^GFAPsgp130^WSD offspring displayed a catch-up growth with increased body weight and egWAT percentage compared to their respective SD controls (Figure 2c,d). Interestingly, P21 egWAT percentage was significantly higher in WSD offspring compared to ^GFAPsgp130^WSD offspring. Both body weight and egWAT at P21 were significantly influenced by maternal diet and genotype as well as their interaction. Additionally, µCT measurements revealed increased amounts of visceral and total body fat in WSD and ^GFAPsgp130^WSD offspring to the respective SD offspring at P21; however, there was no significant difference between WSD and ^GFAPsgp130^WSD (*p* = 0.0827) (Figure 2e).

**Figure 3:**
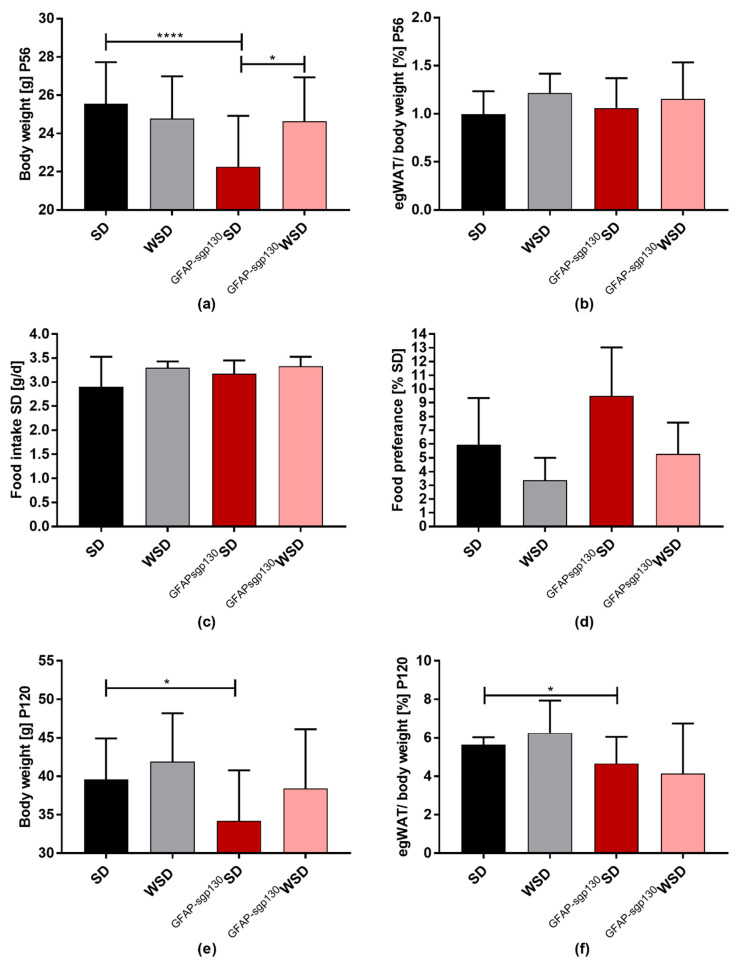
Phenotyping of male offspring at P56 and P120. Data are presented as mean + SD; * *p* < 0.05; **** *p* < 0.0001. (**a**) body weight at P56. *n* = 33–90/group; (**b**) egWAT percentage of mal offspring at P56. *n* = 10–28/group; (**c**) determination of standard diet (SD) food intake at P60. *n* = 5–19/group; (**d**) food preference test at P60 comparing preference for standard diet (SD) or western-style diet (WSD) when offered both diets. SD preference displayed in%. *n* = 7–13/group; (**e**) body weight at P120; *n* = 19–40/group; (**f**) egWAT percentage at P120. *n* = 11–22/group.

At P56, representing adolescence in the offspring [41], there was no significant difference between SD and WSD offspring, while ^GFAPsgp130^WSD offspring was significantly heavier than ^GFAPsgp130^SD offspring (Figure 3a). The maternal genotype and the interaction of maternal genotype and diet showed a significant overall effect. There was no significant difference in egWAT percentage detectable (Figure 3b). At P120, indicating adulthood [41], maternal diet and genotype showed a significant influence determined by two-way ANOVA. Offspring body weight in ^GFAPspg130^SD offspring was significantly decreased compared to SD offspring, but there was no significant difference between ^GFAPspg130^WSD and WSD offspring (Figure 3e). Furthermore, at each time point (P1, P21, P56, P120), ^GFAPsgp130^SD offspring was significantly lighter than SD offspring—an effect not seen when comparing WSD and ^GFAPsgp130^WSD offspring (Figure 2a–c and Figure 3a,e,f).

Additionally, we assessed daily food intake and food preference at P60 to regard whether maternal diet influences the development of taste in offspring. There was no difference in caloric intake detectable between groups (Figure 3e) and no significant difference in food preference (Figure 3f), even though maternal diet and genotype showed significant influence shown by two-way ANOVA. All things considered, we detected a pronounced effect of the maternal diet on body weight and egWAT percentage at P21 in both WSD and ^GFAPsgp130^WSD offspring. In WSD offspring, we detected the highest egWAT percentage at P21 and P120 compared to all other groups, but overall brain-restricted inhibition of IL-6tS had little impact on offspring body weight and egWAT percentage. Of particular interest, the effects of maternal diet on offspring body weight seemed to be independent of caloric intake.

### 3.3. Effects of Maternal Diet Outweight Effects of Brain Restricted Inhibition of IL-6tS on Metabolic Serum Parameters and ipITT in the Offspring at P21

**Figure 4:**
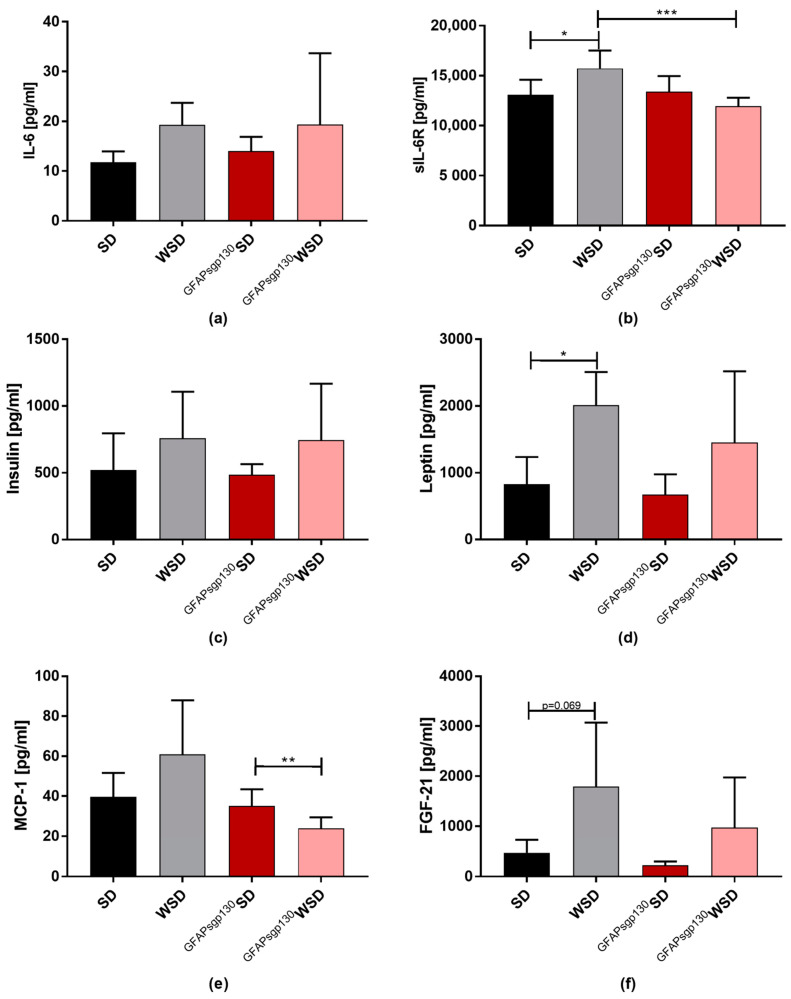
Offspring serum markers at P21. Data are presented as mean + SD or mean; * *p* < 0.05; ** *p* < 0.01; *** *p* < 0.001. (**a**) IL-6 serum concentration. *n* = 6–8/group; (**b**) sIL-6R serum concentration. *n* = 6–7/group; (**c**) insulin serum concentration. *n* = 6–8/group; (**d**) leptin serum concentration. *n* = 6–8/group; (**e**) MCP-1 serum concentration. *n* = 6–8/group; (**f**) FGF-21 serum concentration. *n* = 6–8/group.

To further investigate the effects of maternal diet on offspring metabolism, offspring serum was tested for metabolic and LGI markers at P21. While serum IL-6 levels only tendentially increased in WSD offspring compared to SD offspring (Figure 4a), there was a significant increase in sIL6-R levels in WSD to SD offspring at P21. Interestingly, sIL-6R serum levels in ^GFAPsgp130^WSD offspring were markedly reduced compared to WSD offspring. The interaction of maternal genotype and diet, as well as maternal genotype alone, was significant for sIL-6R (Figure 4b). There was no significant difference in serum insulin levels detectable (Figure 4c). Serum leptin concentrations were significantly increased in WSD offspring compared to SD offspring, while only a tendency of elevation was seen in ^GFAPsgp130^WSD offspring compared to ^GFAPsgp130^SD offspring (Figure 4d). Maternal genotype showed significant two-way ANOVA results. Serum MCP-1 concentration was markedly reduced in ^GFAPsgp130^WSD compared to ^GFAPsgp130^SD offspring, while WSD offspring showed the highest MCP-1 serum concentrations of all four groups (Figure 4e). Here, the interaction of maternal diet and genotype, as well as maternal genotype, were significantly altered as calculated by two-way ANOVA. There was a higher tendency in fibroblast growth factor 21 (FGF-21) serum concentrations in WSD to SD (Figure 4f), and maternal diet showed a significant effect.

**Figure 5:**
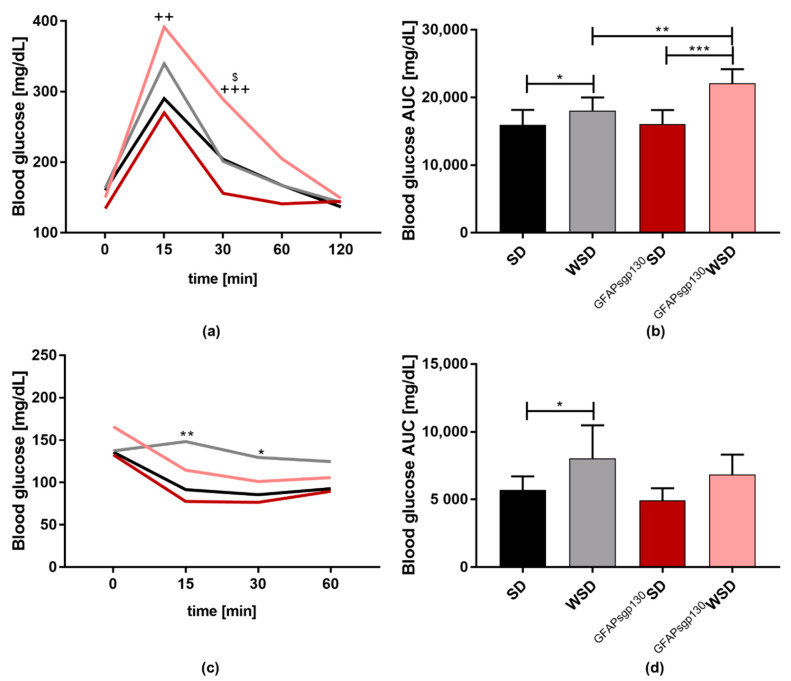
Offspring intraperitoneatl glucose (ipGTT) and insulin tolerance testing (ipITT) at P21. Data are presented as mean + SD or mean; */$ *p* < 0.05; **/++ *p* < 0.01; ***/+++ *p* < 0.001;. In line graphs, * and ** show significances in-between SD and WSD, ++ and +++ show significances in-between ^GFAPsgp130^SD and ^GFAPsgp130^WSD, and $ shows significances between ^GFAPsgp130^WSD and WSD. (**a**) ipGTT and (**b**) area under the curve (AUC) at P21. *n* = 5–10/group; (**c**) ipITT and (**d**) area under the curve (AUC) at P21. *n* = 5–10/group.

At P21, ipGTT in the offspring showed higher blood glucose levels in both WSD groups of offspring compared to their respective SD groups and ^GFAPsgp130^WSD had a higher AUC than WSD offspring (Figure 5a,b). Likewise, two-way ANOVA revealed a significant influence of maternal diet, genotype, and their interaction. During ipITT, WSD offspring was significantly less insulin sensitive than SD offspring (Figure 5c,d) with significant influence of maternal genotype, diet, and their interaction. For WT offspring independent of maternal diet, fasting blood glucose was 156 mg/dL, and in transgenic offspring, fasting blood glucose was 142 mg/dL. Mean ± standard variation for line graphs is provided in Supplementary Table S5.

### 3.4. Effects of Maternal Obesity and Brain Restricted Inhibition of IL-6tS on Offspring egWAT mRNA Expression of Adipokines and egWAT Metabolism

To analyze potential effects of brain-restricted inhibition of IL-6tS on egWAT metabolism via a postulated “hypothalamus-fat-axis”, egWAT mRNA expression of several metabolic key markers was determined at P21.

**Figure 6:**
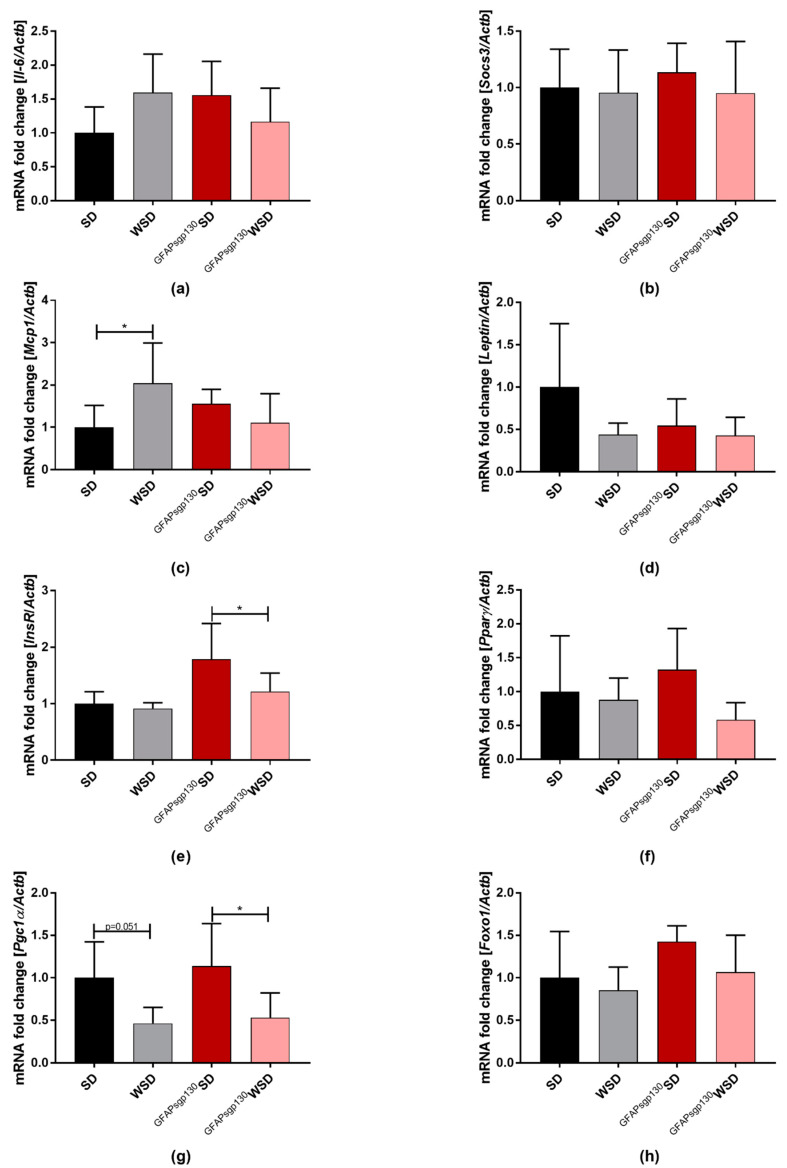
Relative egWAT mRNA expression at P21. Data are presented as mean + SD. SD group is set to 1 to show fold change to SD of other groups. *n* = 8/group; * *p* < 0.05. (**a**) *Il-6*; (**b**) *Socs3*; (**c**) *Mcp-1*; (**d**) *Leptin*; (**e**) *InsR*; (**f**) *Pparγ*; (**g**) *Pgc1α*; (**h**) *Foxo1*.

EgWAT mRNA expression of *Il-6* and *Socs3* was unaltered between groups (Figure 6a,b), even though the interaction of maternal diet and genotype was significant for IL-6 expression (*p* = 0.0082). *Mcp-1* mRNA expression was significantly increased in WSD offspring compared to SD offspring, while ^GFAPsgp130^WSD offspring showed a tendency towards reduced expression compared to ^GFAPsgp130^SD offspring (Figure 6c), and a significant influence of the interaction of maternal genotype and diet was observed (*p* = 0.0045). The assessment of egWAT *Leptin* mRNA expression revealed no significant differences between groups (Figure 6d) with significant influence of maternal diet by two-way ANOVA analysis, but insulin receptor (*InsR*) mRNA expression was significantly influenced by maternal diet and genotype and showed a reduction in ^GFAPsgp130^WSD offspring compared to ^GFAPsgp130^SD offspring, while WSD and SD offspring did not differ (Figure 6e). The same trend was notable for peroxisome proliferator-activated receptor gamma (*Ppar*γ) mRNA expression (Figure 6f), which showed significant influence by maternal diet only. For peroxisome proliferator-activated receptor gamma coactivator 1-alpha (*Pgc1α*), there was a significant reduction detectable in ^GFAPsgp130^WSD to ^GFAPsgp130^SD and a tendency in WSD offspring compared to SD offspring (Figure 6g) and a significant influence by maternal diet (two-way ANOVA). Forkhead box protein O1 (*Foxo1*) mRNA expression at P21 was not significantly affected (Figure 6h) but showed significant influence by maternal genotype.

### 3.5. Effects of Maternal Obesity and Brain-Restricted Inhibition of IL-6tS on the Offspring’s egWAT Proteome at P21

**Figure 7:**
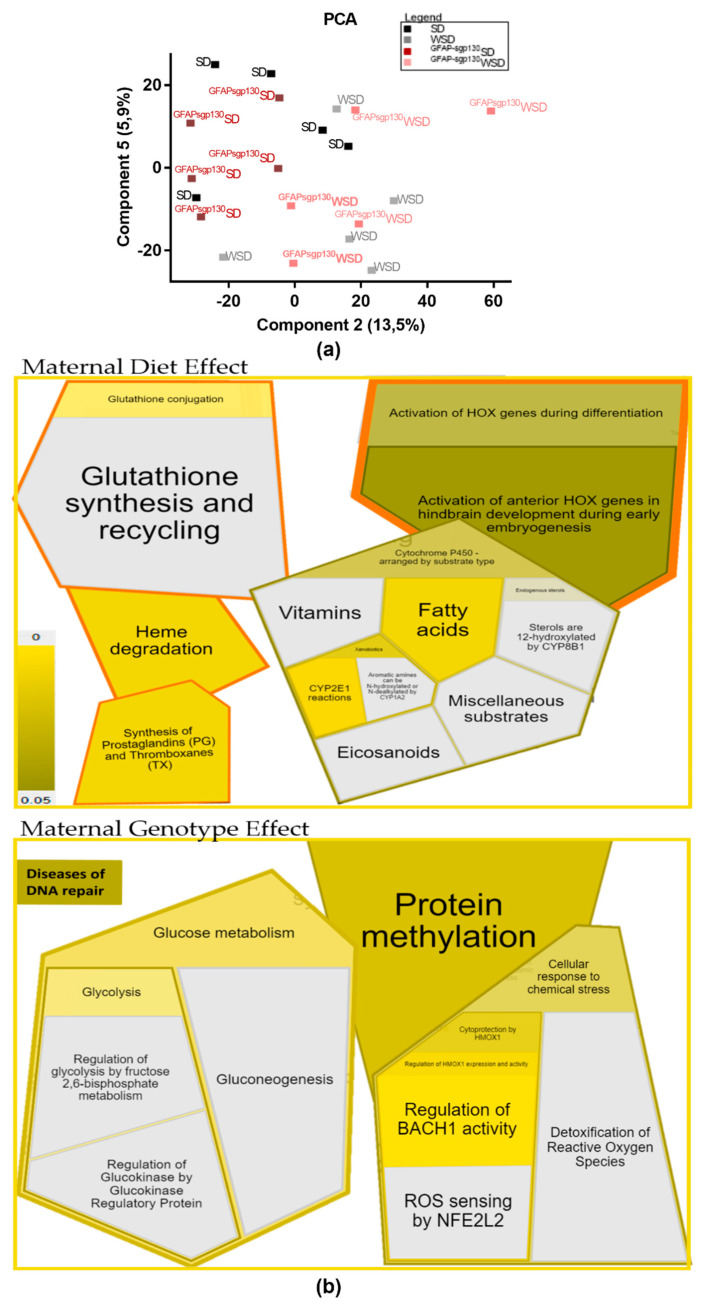
Epigonadal white adipose tissue (egWAT) proteomics data in a four-group comparison. Two -way ANOVA is calculated in Perseus and Bonferroni-corrected. *n* = 5/group (**a**) principal component analysis (PCA); (**b**) gene list analysis of protein differentiated by maternal diet and maternal genotype adapted from reactome.org. Significantly regulated pathways are marked yellow (for color legend, see left-hand side).

To further assess the effects of brain-restricted IL-6tS on a potential “hypothalamus-fat-axis” in the offspring, we additionally performed proteome analysis in offspring egWAT at P21. In total, 6160 proteins were identified, and a principal component analysis (PCA) was generated, with component 2 explaining 13.5% and component 5 explaining 5.9% of the total clustering, which showed slight differences between all four groups of offspring (Figure 7a). Components 2 and 5 were chosen to focus on components explaining less pronounced differences in-between the groups on global egWAT protein expression. Component 2 separates groups coarsely due to maternal diet, showing a slight right-shift of both WSD offspring groups. Furthermore, using a two-way analysis and Bonferroni correction, 1 protein was differentially expressed due to maternal diet and genotype, 4 proteins were differentially expressed due to maternal diet, and 12 proteins were differentially expressed due to maternal genotype (see Supplementary Table S6). Proteins that differed by maternal diet were subjected to reactome.org version 77 and showed an involvement mainly in metabolic processes, amongst these biological oxidations, such as fatty acid oxidation and CYP2E1 reactions, as well as fatty acid metabolism, in specific the synthesis of prostaglandins and thromboxanes. Furthermore, heme degradation, HOX genes, and glutathione synthesis were significantly influenced by maternal diet. Proteins that differed by maternal genotype were likewise analyzed with reactome.org and revealed, amongst others, protein methylation, diseases of DNA repair, glucose metabolism, and cellular response to chemical stress, mainly influenced by the regulation of Bach1 activity (Figure 7b). A list of significantly altered pathways by maternal diet and genotype is provided (Supplementary Table S7).

## 4. Discussion

In this study, we aimed at characterizing the effects of maternal obesity on the offspring’s energy metabolism and egWAT function and examined whether brain-restricted IL-6tS inhibition can partially prevent detrimental effects of maternal WSD feeding on offspring. We subjected transgenic ^GFAPsgp130^mice with a brain-restricted IL-6tS inhibition to maternal obesity before pregnancy, during pregnancy, and lactation and compared them to WT controls undergoing the same protocol.

Before pregnancy, transgenic ^GFAPsgp130^dams were generally lighter than WT animals, and transgenic dams showed delayed body weight gain. The breeding strategy for the transgenic animals in our study did not account for crossbreeding with WT animals, so we cannot determine whether the difference in body weight is a specific result of brain-restricted inhibition of IL-6tS. Escrig et al. analyzed the effects of ^GFAPsgp130^mice in the context of maternal high-fat diet (HFD) and Alzheimer’s disease. In their study, in which they performed crossbreeding, the bodyweight of 17-month-old male and female offspring was consistently lower than the respective control group, but not to a significant extent [42]. Taking into account that Escrig et al. showed similar observations, effects based on the lack of crossbreeding are unlikely.

As expected from previous experiments by our group and others [43,44], WSD and ^GFAPsgp130^WSD dams displayed signs of impaired glucose tolerance prior to mating. Importantly, entering pregnancy, transgenic and WT dams on WSD were significantly heavier than the respective SD groups with no significant differences in-between WT and transgenic dams on the same diets, marking no significant effect of body weight differences entering the perinatal period. Thus, the experimental design proved to be valid and provided an equally affected perinatal environment for the respective offspring.

In the offspring, maternal WSD caused an increase in body weight and egWAT percentage up to P21 in both WT and transgenic offspring, indicating a catch-up growth when compared to the respective bodyweights at P1 [45]. Interestingly, egWAT mass was clearly reduced in ^GFAPsgp130^WSD offspring compared to WSD offspring, while µCT analysis could not show significant effects in egWAT, possibly due to a low number of replicates. However, at later time points (P56 and P120), no clear pattern of protection or reversal of phenotypic features (body weight, egWAT percentage or glucose tolerance) was detectable upon brain-restricted IL-6tS inhibition.

Throughout P21, P56, and P120, ^GFAPsgp130^SD offspring reveal lower body weights than SD offspring. Possibly, this can be explained by the brain-restricted IL-6tS inhibition. The role of neurogenesis in mice on body weight homeostasis is supported by the literature [46,47]. Campbell et al. could show that brain-restricted IL-6tS inhibition significantly reduced astrogliosis and that brain-specific inflammation induced by GFAP-IL6 mice is linked to altered neurogenesis and gliosis, but did not regard body weight [14]. Additionally, as mentioned above, Escrig and colleagues also observed a lower body weight in ^GFAPsgp130^mice on a standard diet. Even if the lower body weight was not to a significant extent, this effect was also blunted after feeding an HFD [42]. In line with Escrig et al., food intake in male mice was not altered by centrally blocking IL-6tS. Brain-restricted IL-6tS might therefore reduce body weight, provoking changes in neurogenesis in transgenic mice on SD. The lack of the permanent effect in ^GFAPsgp130^WSD mice is possible because the unfavorable WSD-mediated effects on the whole-body metabolism outweigh the effects of brain-specific IL-6tS inhibition to reduce body weight. Therefore, the reason for the reduced body weight in ^GFAPsgp130^SD offspring remains not fully understood and should be addressed in future studies.

Previous research found that taste development is influenced prenatally [48]. As taste preference of high caloric food would encourage the individual to consume a diet that will induce obesity, we evaluated food preference to establish whether maternal diet had a measurable impact on taste in offspring and if IL-6tS inhibition in the brain would change this. The food preference test showed no significant priming of offspring’s taste development by the maternal diet, and no influence of IL-6tS was observed.

Since circulatory MCP-1 has been linked to monocyte recruitment during inflammation as well as LGI [49], the data suggest less inflammation in egWAT of ^GFAPsgp130^WSD offspring. Kraakman et al. underline the effect of IL-6tS as an advocate of adipose tissue macrophage recruitment, in which MCP-1 also plays a vital role [18]. Reduced levels of MCP-1 were shown to support an elevation in energy expenditure in adipose tissue through an elevation of browning [18,50]. While Kraakman et al. used a model of peripheral inhibition of IL-6tS, our data indicate that brain-restricted IL-6tS inhibition has an impact on MCP1 serum levels and MCP1 mRNA levels in egWAT as well. A confirmation by immunohistologic staining of egWAT regarding macrophage recruitment will be helpful to understand the mechanism better.

The sIL-6R was correlated with T2D. Kado et al. revealed that patients suffering from T2D had elevated plasma sIL-6R concentrations [51]. In plasma samples of T2D patients, Kraakman et al. established that concentration of sIL-6R positively correlated with HOMA-IR, HbA1c, and body mass and concluded that the sIL-6R is a disease marker for T2D. In our study, WSD offspring at P21 presented elevated body weight, body fat content, sIL-6R serum levels, and impaired glucose tolerance compared to the respective SD offspring, supporting the findings of Kraakman et al. and Kado et al., underlining the importance of maternal obesity on offspring glucose homeostasis. Interestingly, sIL-6R concentration in serum was markedly lower in ^GFAPsgp130^WSD compared to WSD offspring. In contrast, others reported a reduction in sIL-6R in T2D compared to healthy controls [52]. Thus, neither an elevation nor a reduction in sIL-6R serum levels seems to be linked to an improved glucose sensitivity. It is reasonable that normal-ranged sIL-6R levels are necessary for adequate glucose homeostasis.

The candidate gene approach to assess egWAT metabolism mostly confirmed the phenotyping results at P21: *Mcp-1* mRNA expression showed elevation due to maternal WSD only in WT offspring, while all markers involved in insulin sensitivity and action (*InsR*, *Ppar*, *Pgc1α* and *Foxo1*) showed the same trends and effects in both, WT and transgenic offspring. Interestingly, there was no significant effect of either maternal obesity or brain-specific inhibition of IL-6tS on egWAT *Il-6* and *Socs3* mRNA expression detectable in post-hoc tests, even though the interaction of the variables shows the significant influence on mRNA *Il-6* expression. This is in contrast to previous findings from our group. In 2016, we reported that maternal diet-induced obesity leads to increased serum IL-6 concentrations and upregulated hypothalamic and egWAT IL-6 signaling in the offspring at P21 [20]. However, the diet used in these previous experiments differed from the diet used in this study. While the previous HFD contained 60% fat of total metabolizable energy [44], the current WSD for induction of obesity in the dams was potentially more harmful, as it contained 45% fat as well as 19.1% sugar of total metabolizable energy [53]. Here, we chose a WSD to more accurately represent the obesogenic diet pattern in humans [54]. Gao et al. showed a higher impact of high sugar consumption on hypothalamic inflammation [55], while others have examined inflammatory effects due to both high fat and high sugar consumption [56,57]. All groups on HFD or WSD displayed impaired glucose tolerance during ipGTT. However, maternal absolute weight gain at mating on HFD was 5.27 g [8], while WSD only induced a weight gain at the mating of 3.84 g in wild type and 3.49 g in transgenic mice. Thus, the lack of elevated IL-6 serum concentrations in WSD dams might be due to the unexpected lower weight gain on WSD when compared to HFD. Clinical data on IL-6 serum concentrations in children of obese mothers are scarce. Interestingly, clinical studies on IL-6 serum concentrations in pregnant women also report conflicting results of maternal obesity: while quite a number of studies report a positive correlation between IL-6 levels and maternal obesity, other studies cannot confirm this [58]. Future studies are needed to clarify the exact contribution of dietary components and maternal factors on maternal and offspring’s circulating IL-6.

We chose an unbiased approach to determine any effects of maternal obesity and nutrition and brain-restricted inhibition of IL-6tS on egWAT in the offspring at P21. While the use of Bonferroni-corrected two-way ANOVA limits false-positive results, a higher false-negative rate must be taken into account that might blunt effects of IL-6tS inhibition on WSD effects in offspring. To the best of our knowledge, the present data provide the first proteomic approach comparing the effects of maternal obesity on egWAT function in offspring. Analysis of the egWAT proteome revealed only a few proteins that were significantly regulated by maternal diet or genotype.

Regarding the cluster showing proteins depended on maternal diet, a change in “CYP2E1 reaction” is visible. “CYP2E1 reactions” have previously been linked to obesity-induced oxidative stress [59]. In the liver, CYP2E1 concentrations are strongly linked to morbidly obese subjects and elevated further in patients with steatosis while showing a decrease due to weight loss [60]. In rats, Yoshinari et al. were able to show a concomitant regulation of CYP2E1 in the liver and WAT [61]. The pathway “heme degradation” marks the breakdown of heme by heme-oxygenase (HO). HO acts to reduce obesity-induced inflammation, and decreased HO activity results in increased inflammatory states such as hypertension [62].

Moreover, neuroendocrine regulations are known to be affected by the fatty acid composition of cell membranes [63], emphasizing the impact of different “fatty acids” observed due to maternal diet. “Glutathione synthesis and recycling” also differed by maternal diet. Obesity itself is linked to enhanced oxidative stress, and glutathione is involved in reducing oxidative stress [64]. Glutathione content is altered by its antioxidant activity induced in the adipose tissue of rodents introduced to oxidative stress [65]. The impairment of glutathione metabolism is linked to the obese state and disrupted insulin signaling [66]. Interestingly, maternal obesity has been linked to lower antioxidant enzyme capacity in the placenta, and those mothers carrying male offspring show the highest amount of oxidative stress in the placenta [67]. The results of the proteomic analysis support these findings and mark significantly more oxidative stress due to maternal obesity.

“HOX genes activation during differentiation” shows significant alterations due to maternal diet in egWAT proteomic analysis. HOX genes are associated with the transcriptional regulation of adipogenesis in humans [68], and alteration of HOX genes have been linked to metabolic defects [69]. Alteration in HOX genes due to diabetes was also observed in rodent lungs [69]. Besides, HOX genes are known to be expressed differently in different adipose tissue deposits as well as adipocyte types. Brown adipose tissue shows differentiated HOX gene expression than white adipose tissue [68]. In combination with results drawn from differed MCP-1 expression in serum and mRNA analysis, a higher beiging turnover of egWAT adipocytes is probable in offspring not affected by maternal obesity. This mechanism might be part of the reason why offspring from SD and ^GFAPsgp130^SD dams attain less egWAT percentage and should be addressed in future studies. “Prostaglandin synthesis” is significantly altered in offspring’s egWAT by maternal diet. Higher prostaglandin production is present in obesity, and it is likely to be ascribed to elevated circulating free fatty acids in offspring that were challenged with maternal obesity [70]. Strong associations in prostaglandin synthesis and TNFα and MCP-1 expression in HFD-induced obese, insulin-resistant rats were observed by Chan et al. and inhibition of prostaglandin receptor diminished inflammatory gene and protein expression [71]. Thus, future experiments are required to clarify if different prostaglandin synthesis due to maternal diet is likely linked to passing on the risk of the development of adipose tissue inflammation.

Due to our stringent approach and Bonferroni correction after two-way ANOVA, there are no significantly altered proteins influenced by the interaction of maternal diet and genotype. However, protein expressions influenced by maternal genotype independent of maternal diet describe pathways that are altered by brain-restricted IL-6tS inhibition and not affected by potentially detrimental consequences of maternal obesity. Selected pathways significantly altered by maternal genotype are “protein methylation”, “glucose metabolism, “glycolysis” and “cellular responses to stress by regulation of BACH1 activity”. Here, we have limited knowledge of whether these alterations are solemnly due to IL-6tS inhibition in the brain or due to the mentioned lack of crossbreeding. Keeping this limitation in mind, diet-independent alterations of the pathway “protein methylation” in offspring egWAT might indicate modifications in the offspring’s egWAT by brain-restricted IL-6tS inhibition. Epigenetic changes play an important role in the regulation of activation and inhibition of RNA and consequently proteins [72], and especially methylation as one possible epigenetic change, is discussed to be an important player in perinatal programming [73]. Future studies should address methylation analysis to clarify the influences of centrally blocking IL-6tS on offspring egWAT metabolism.

The differentially altered pathways “glucose metabolism” and “glycolysis” by maternal genotype potentially give insight to a differentiated glucose processing in egWAT of transgenic versus WT offspring [74]. Moreover, our experiments revealed the highest blood glucose levels for ^GFAPsgp130^WSD offspring compared to WSD at P21. This is in accordance with findings by colleagues. Escrig et al. revealed higher blood glucose levels in female ^GFAPsgp130^SD to SD mice, an effect that was also visible in male ^GFAPsgp130^mice [42]. This finding is supported by Timper et al., who revealed that central injection of IL-6 improved hepatic insulin action and glucose tolerance in obese mice [75]. In addition, peripheral inhibition of IL-6tS revealed no influences on glucose sensitivity in standard or HFD-fed mice [18]. In summary, brain-restricted IL-6 inhibition does not seem to be beneficial for whole-body glucose homeostasis and has even worse effects when mice are exposed to a WSD.

As for limitations in our study, only male offspring were examined, predominantly for reasons of available resources and time, but also due to higher variability in female offspring stated by others [76,77,78]. It is equally important to understand mechanisms involved in female offspring to detect sex-specific differences. Our approach was to first unravel mechanisms in male offspring as a more simplistic model and then further assess alteration in female offspring in the further course. Since several published studies examining the effects of maternal obesity on the offspring used male offspring only [79,80,81,82], with the current literature stating the same observation [83], we started by analyzing male offspring to make our data comparable with those studies.

Although the translation of findings from rodent studies to humans is difficult and should only be performed with care, our results clearly underline the importance of education and intervention programs for obese pregnant women to reduce the vast array of complications for generations to come.

In conclusion, our study used a straightforward approach to determine the potential effects of brain-restricted IL-6tS inhibition on offspring short- and long-term metabolism and offspring egWAT protein expression at the end of lactation. While we observed strong effects of maternal diet and obesity on the offspring’s phenotype in early life, thorough phenotyping and unbiased assessment of global egWAT protein expression revealed few existing differences due to brain-restricted IL-6tS inhibition that are not able to reverse effects of maternal obesity. Furthermore, brain-restricted IL-6tS inhibition even had a non-beneficial impact on glucose homeostasis in the offspring, while inflammatory markers such as MCP-1 were ameliorated. Our data emphasize the importance of maternal nutrition during pregnancy and lactation on the offspring’s short and long metabolism.

## Data Availability

The data presented in this study are available on request from the corresponding author.

## Supplementary Materials

The following are available online at www.mdpi.com/article/10.3390/nu13113735/s1, Figure S1: Litter size. Table S1: Information on experimental diets used. Table S2: Primers for mRNA analysis. Table S3: Mean ± standard deviation for all line graphs in Figure 1. Table S4: Mean ± standard deviation for all line graphs in Figure 2. Table S5: Mean ± standard deviation for all line graphs in Figure 5. Table S6: Proteomic analysis with Bonferroni-corrected two-way ANOVA. *N* = 5/group. Table S7: Pathways significantly regulated by maternal diet, analyzed with reactome.org version 77. Supplementary Table S8: Pathways significantly regulated by maternal genotype, analyzed with reactome.org version 77.
