# Supplementary material for: Brain-Restricted Inhibition of IL-6 Trans-Signaling Mildly Affects Metabolic Consequences of Maternal Obesity in Male Offspring
Source: Nutrients. 2021 Oct 23;13(11):3735. doi: 10.3390/nu13113735 (PMC8618896; doi:10.3390/nu13113735)
Supplement: Supplementary file 1 [file nutrients-13-03735-s001.zip › Supplementary Material.pdf]

Appendix A

Animal procedures

Animals are kept in in individually ventilated cages (IVCs, Blue line cages type II long, Tecniplast, Italy) at the animal facility of the Department of Pharmacology of the University Hospital of Cologne (Cologne, Germany) in one consistent room in two racks holding 6x5 or 7x5 cages, respectively. Mice are kept separately in groups of 3 to 5. They are only separated if necessary for experimental purposes. Pregnant mice are single-housed to ensure assignability of pups to dams. Male rams used for breeding are kept single to prevent hierarchic encounters. The room temperature is controlled to 22 °C and a humidity of 50-60% and a 12/12-h light/dark cycle. The cages, food pellets and water supply are changed once weekly by the assigned animal care attendants. Experiments are carried out during the daytime. Weight progression measurements are noted in the mornings between 6:00 am and 8:00 am. Experiments with a mandatory fasting period before execution are carried out at noon between 1:00 pm and 3:00 pm, starting the fasting period at 7:00 am for a 6-hour fast. Animals are handled with rubber gloves to avoid contact with an external atmosphere. Cages are only opened under a hood to deduct possible contamination. Female mice grouped as dams are only mated once to produce offspring. Male rams are mated repetitively starting at 10 weeks of age. For mating, one female mouse is placed into the cage of a male ram for olfactory purposes. The couple is mated for three consecutive days. The female dam is weighed on gestational day 1 (G1) and again at G3 during the removal of the ram. The weight progression is traced by measurements every other day on weekdays until bearing. Litter size and sex distribution is noted. Litter size is reduced to n=6 pups per dam on postnatal day 3 (P3), consisting of 4 male and 2 female offspring. Pups are reduced by decapitation. If the litter does not account for the desired distribution, the closest distribution possible is chosen. A litter of n=5 is substituted with an excess pup of another dam from the same genetic background and diet group born on the same day. If the weighing day falls on the weekend, the mean of the bordering measurements is used. Female mice on SD are mated if their body weight does not exceed 23 g while female mice on WSD are bred with a body weight above 23 g to ensure their response to WSD.

Appendix B

Supplemental Figure S1: Litter size. n=64-130/group.

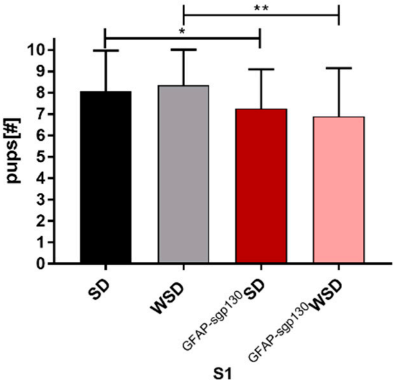

Supplementary Table S1: Information on experimental diets used.

Diet			**SD**	**WSD**
Name			**complete feed for rats & mice**	**DIO – 45 kJ% fat (Lard)**
Company			Ssniff	Ssniff
Order number			V1534 - R/M-Maintenance	E15744-344
Additional information			complete feed for rats & mice	HF diet for rodents with lard (& soybean oil)
Energy (metabolizable)		kcal/kg	3225	4615
Energy(metabolizable)		MJ/kg	13,5	19,30
Gross Energy		MJ/kg	16,2	22,50
Fat		kJ%	9	45,00
Protein		kJ%	24	20,00
Carbohydrates		kJ%	67	35,00
Of that sugar		kJ%	8,8	19,1
Crude Fat		g/kg	33	236
Crude Protein		g/kg	190	220
Crude Fibre		g/kg	50	57
Crude Ash		g/kg	64	53
N free extracts		g/kg	546	400
**Sugar**				
Sucrose		g/kg	-	211,0
Monosaccharides		g/kg	54,0	-
Dissacharides		g/kg		-
Sugar (total)		g/kg	54	211,0
**Carbohydrates**				
Dextrin		g/kg	-	108,0
Polysaccharides		g/kg	359	-
Starch		g/kg	-	68

Carbohydrates (total)		g/kg	413	387
Cellulose powder		g/kg	-	57
Cholesterol		mg/kg	-	-
**Fat sources**				
Soybean oil		%	n/a	2,8
Butter fat		%	n/a	-
Corn oil		%	n/a	-
Pork lard		%	n/a	20,8
Palm oil		%	n/a	-
**Fatty acids**				
Butanoic acid	C 4:0	mg/kg	-	-
Caproic acid	C 6:0	mg/kg	-	-
Caprylic acid	C 8:0	mg/kg	-	-
Capric acid	C-10:0	mg/kg	-	-
Lauric acid	C-12:0	mg/kg	-	500,00
Myristic acid	C-14:0	mg/kg	0,01	2900,00
Palmitic acid	C-16:0	mg/kg	0,45	53300,00
Margaric acid	C-17:0	mg/kg	-	-
Stearic acid	C-18:0	mg/kg	0,09	29200,00
Arachidic acid	C-20:0	mg/kg	0,01	70,00
Palmitoleic acid	C-16:1	mg/kg	0,01	6200,00
Oleic acid	C-18:1	mg/kg	0,62	94200,00
Linoleic acid	C-18:2	mg/kg	1,66	34600,00
Alpha-linolenic acid	C-18:3	mg/kg	0,23	3700,00
Eicosanoic acid	C-20:1	mg/kg	-	-
Eicosadienoic acid	C-20:2	mg/kg	-	-
Arachidonic acid	C-20:4	mg/kg	-	-
**Minerals**				

Calcium		g/kg	10,00	92,00
Phosphorus		g/kg	7,00	64,00
Magnesium		g/kg	2,20	23,00
Sodium		g/kg	2,40	20,00
Potassium		g/kg	9,10	97,00
Sulfur		g/kg	n/a	n/a
Chlorine		g/kg	n/a	n/a
Ca/P		%	1,43:1	1.44:1
**Trace Elements**				
Iron		mg/kg	189	168
Manganese		mg/kg	68	95
Zinc		mg/kg	91	65
Copper		mg/kg	15	13
Iodine		mg/kg	2,1	1,2
Molybdenum		mg/kg	-	n/a
Fluorine		mg/kg	-	n/a
Selenium		mg/kg	0,3	0,2
Cobalt		mg/kg	-	n/a
Mineral & trace element mix		%	n/a	6
**Vitamins**				
Vitamin A		I.E./kg	15.000	15000
Vitamin D3		I.E./kg	1.100	1500
Vitamin E		mg/kg	110	150
Menachinone		mg/kg	7	20
Thiamin		mg/kg	18	25
Riboflavin		mg/kg	22	16
Vitamin B6		mg/kg	21	16
Vitamin B12		mg/kg	0,1	30

Nicotinic acid		mg/kg	115	47
Pantothenic acid		mg/kg	40	55
Folate		mg/kg	7	16
Biotin		mg/kg	0,51	300
Choline		mg/kg	1370	920
Choline chloride		mg/kg	not added	0,2
Vitamin premix		%	not added	1
**Amino acids**				
Lysine		mg/kg	10000	18300
Methionine		mg/kg	3300	7800
Cystine		mg/kg	3500	3900
Met+Cys		mg/kg	6800	11800
Threonine		mg/kg	7100	9700
Tryptophan		mg/kg	2500	2900
Arginine		mg/kg	11900	8600
Histidine		mg/kg	4400	6700
Valine		mg/kg	9000	15400
Isoleucine		mg/kg	7900	12500
Leucine		mg/kg	13900	21900
Phenylalanine		mg/kg	8800	11400
Phe+Tyr		mg/kg	14900	23100
Glycine		mg/kg	8800	4700
Glutamic acid		mg/kg	41000	49700
Aspartic acid		mg/kg	17900	16400
Proline		mg/kg	12900	25300
Serine		mg/kg	9900	13200
Alanine		mg/kg	8200	6600

Supplementary Table S2: Primers for mRNA analysis.

Primer	Sequence (5′-3′)
β-Actin	Forward, TGACAGGATGCAGAAGGAGATTACT
	Reverse, GCCACCGATCCACACAGAGT
	Probe (FAM), ATCAAGATCATTGCTCCTCCTGAGCGC (TAMRA)
Gapdh	Forward, ATGTGTCCGTCGTGGATCTGA
	Reverse, TGCCTGCTTCACCACCTTCT
	Probe (FAM), CCGCCTGGAGAAACCTGCCAAGTATG
18S	Forward, AGATCCCAGACTGGTTCCTG
	Reverse, TTGTTGTCTAGACCGTTGGC
	Probe (FAM), CAGAACCTGGCTGTACTTCCCATCC
IL-6	Forward, ACAAGTCGGAGGCTTAATTACACAT
	Reverse, AATCAGAATTGCCATTGCACAA
	Reverse, CTTTTCTCATTTCCACGATTTCCCAGAGAA
Socs3	Forward, CCACCCTCCAGCATCTTTGT
	Reverse, TCCAGGAACTCCCGAATGG
	Probe (FAM), ACTGTCAACGGCCACCTGGACTCCT
MCP-1	Forward, GGCTCAGCCAGATGCAGTTAAC
	Reverse, CTTGGTGACAAAAACTACAGCTTCTT
	Probe (FAM), CCCCACTCACCTGCTGCTACTCATTCA
Leptin	Forward, TCACCAGGATCAATGACATTTCAC
	Reverse, AGCCCAGGAATGAAGTCCAA
	Probe (FAM), ACGCAGTCGGTATCCGCCAAGC
InsR*	Forward, GCAAACAGATGCCACTAATCCTT
	Reverse, GGGCTTCCACTTTAAGATAATCTGA
PPARγ	Forward, CCCAATGGTTGCTGATTACAAA
	Reverse, GCCTGTTGTAGAGCTGGGTCTT

	Probe (FAM), ACCTGAAGCTCCAAGAATACCAAAGTGCGA
PGC1α	Forward, TCGAAAAAGAAGTCCCATACACAA
	Reverse, TTCCACACTTAAGGTTCGCTCAATA
	Probe (FAM), CACCAAATGACCCCAAGGGTTCCC
Foxo-1*	Forward, CAATGGCTATGGTAGGATGG
	Reverse, TTTAAATGTAGCCTGCTCAC

*Genes were measured by the SYBR-Green method. No probe was needed.

Supplementary Table S3: Mean± standard deviation for all line graphs in Figure 1

Figure 1B: Dam weight progression from week 3 to Gestational Day 19. N=33-58/group

Time	SD		WSD		GFAPsgp130SD		GFAPsgp130WSD	
	mean	SD	mean	SD	mean	SD	mean	SD
Week 3	10.82	1.38	10.98	1.11	9.39	1.77	9.38	1.95
Week 4	15.29	1.76	14.43	2.34	12.66	2.20	13.97	1.65
Week 5	16.98	1.45	16.78	1.70	15.08	1.90	15.90	1.30
Week 6	18.15	1.46	18.22	1.22	16.45	1.57	17.02	1.63
Week 7	19.09	1.50	19.23	1.35	17.30	1.53	17.89	1.53
Week 8	19.92	1.58	20.50	1.45	17.90	1.50	18.88	1.14
Week 9	20.45	1.57	21.38	1.42	18.55	1.42	19.50	1.15
Week 10	21.16	1.29	22.30	1.51	18.87	1.46	20.20	1.24
Week 11	21.38	2.09	23.15	1.46	19.39	1.97	21.44	1.61
Week 12	21.55	1.46	23.92	1.62	19.81	2.05	21.96	1.63
Week 13	21.94	1.61	24.65	1.91	21.02	1.81	22.70	1.93
Week 14	21.58	0.18	26.85	3.79	20.19	1.29	24.05	2.39
Week 15	21.77	0.01	27.06	7.56	20.60	1.01	26.51	1.39
Gestational Day 1	21.90	1.48	25.23	2.72	20.39	1.92	24.66	2.66
Gestational Day 3	22.74	1.52	25.95	2.49	21.00	2.01	24.97	2.76
Gestational Day 5	23.73	2.57	27.09	2.99	21.63	1.95	26.05	2.79
Gestational Day 7	24.32	1.83	28.09	3.21	22.44	1.97	26.25	2.78

Gestational Day 9	25.69	1.85	29.06	3.07	23.40	2.45	27.25	3.00
Gestational Day 11	27.46	2.32	30.30	3.07	24.92	2.42	28.76	3.85
Gestational Day 13	30.02	3.06	32.79	3.41	27.60	2.42	31.18	3.66
Gestational Day 15	33.13	3.63	35.14	4.18	30.55	3.10	33.96	4.03
Gestational Day 17	36.17	3.56	38.86	4.32	32.78	3.54	35.54	3.46
Gestational Day 19	39.47	3.39	42.61	4.49	35.99	4.17	39.27	3.75

Figure 1C: Dam weight progression from week 12 to week 15. N=8-32/group

Time	SD	WSD	^{GFAPsgp130}SD	^{GFAPsgp130}WSD
Week 12	21.66±1.32	23.64±1.51	19.86±2.55	21.01±1.87
Week 13	22.27±1.44	24.07±1.72	21.18±1.64	21.97±2.35
Week 14	22.01±1.11	27.59±5.31	20.80±1.60	22.63±3.02
Week 15	22.29±1.58	30.32±5.97	21.31±1.4	26.06±6.92

Figure 1E: Dam glucose tolerance test. N=10/group

Time	SD	WSD	^{GFAPsgp130}SD	^{GFAPsgp130}WSD
0 min	143±27	176±22	128±15	143±17
15 min	291±41	373±64	245±58	339±38
30 min	221±27	287±43	195±50	269±59
60 min	168±19	199±19	164±21	202±35
120 min	145±16	143±13	139±15	139±20

Figure 1G: Dam insulin tolerance test. N=10/group

Time	SD	WSD	^{GFAPsgp130}SD	^{GFAPsgp130}WSD
0 min	143±27	176±22	128±15	143±17
15 min	291±41	373±64	245±58	339±38
30 min	221±27	287±43	195±50	269±59
60 min	168±19	199±19	164±21	202±35

Supplementary Table S4: Mean± standard deviation for all line graphs in Figure 2

Figure 2A: Offspring Body Weight. N=64-130/group

Time	SD		WSD		GFAPsgp130SD		GFAPsgp130WSD	
	mean	SD	mean	SD	mean	SD	mean	SD
Postnatal Day 1	1.42	0.11	1.4	0.18	1.4	0.20	1.33	0.20
Postnatal Day 3	2.2	2.24	1.95	0.49	1.85	0.44	1.94	0.45
Postnatal Day 5	3.11	0.60	3.01	0.71	2.86	0.72	2.98	0.55
Postnatal Day 7	4.34	0.64	4.26	0.97	3.94	0.89	4.5	0.86
Postnatal Day 9	5.49	0.83	5.44	1.06	4.74	1.04	5.94	1.18
Postnatal Day 11	6.33	0.88	6.5	1.37	5.53	1.08	7.08	1.41
Postnatal Day 13	7.13	0.99	7.53	2.03	6.22	1.17	8.14	1.29
Postnatal Day 15	7.74	0.97	8.62	3.22	6.91	1.13	8.9	1.37
Postnatal Day 17	8.29	0.77	9.32	3.19	7.43	1.21	9.28	1.37
Postnatal Day 19	9.15	0.87	10.48	2.15	8.1	1.36	10.41	2.01
Postnatal Day 21	10.41	1.42	11.98	2.11	9.21	1.61	11.63	1.79
Postnatal Day 28	17.68	2.15	16.3	2.78	14.16	2.86	17.26	2.45
Postnatal Day 35	20.57	1.90	19.18	2.76	17.9	.28	21.29	2.70
Postnatal Day 42	22.52	1.89	21.86	2.28	19.82	.75	22.18	1.88
Postnatal Day 49	24.37	1.79	23.59	2.48	21.3	2.92	23.84	2.35
Postnatal Day 56	25.05	2.66	24.97	2.64	21.97	3.03	24.69	3.68
Postnatal Day 63	27.06	2.22	26.47	2.76	23.6	3.11	25.88	2.61
Postnatal Day 70	28.73	2.87	28.6	3.38	24.91	3.10	29.23	3.86
Postnatal Day 77	30.34	3.04	30.49	4.31	26.61	4.49	32.3	4.83
Postnatal Day 84	32	4.03	32.76	5.28	27.69	5.08	34.05	6.02
Postnatal Day 91	33.46	4.51	35.14	5.48	28.74	5.98	36.38	6.00
Postnatal Day 98	36.81	5.18	37.6	6.27	30.55	6.21	37.36	6.34
Postnatal Day 105	38.14	5.62	39.12	6.75	30.61	6.03	39.41	7.40
Postnatal Day 112	39.6	5.81	40.49	6.22	33.23	6.88	40.38	7.38

Postnatal Day 120	40.02	6.55	41.79	6.80	35.24	7.05	40.33	8.16

Supplementary Table S5: Mean± standard deviation for all line graphs in Figure 5

Figure 5A: Offspring glucose tolerance test. N=5-10/group

Time	SD	WSD	^{GFAPsgp130}SD	^{GFAPsgp130}WSD
0 min	149±29	163±16	134±8	150±16
15 min	272±92	339±82	270±53	391±81
30 min	178±87	201±40	156±10	289±48
60 min	147±42	167±23	141±29	204±23
120 min	140±24	143±29	144±21	149±17

5C: Offspring insulin tolerance test. N=5-10/group

Time	SD	WSD	^{GFAPsgp130}SD	^{GFAPsgp130}WSD
0 min	135+42	137+26	132+15	166+28
15 min	91+23	148+49	77+17	114+32
30 min	85+16	129+56	76+6	101+23
60 min	92+28	124+39	89+15	105+21

Supplementary Table S6: Proteomic analysis with Bonferroni-corrected two-way ANOVA. N=5/group

	**Mean**				**Bonferroni- corrected two-way ANOVA p-value (p=0.00000812)**		
**Protein**	**SD**	**WSD**	**GFAPsgp130 SD**	**GFAPsgp130 WSD**	**Diet**	**Genotype**	**Interaction**
Ajuba	NaN*	NaN*	16.1508	17.7229	**2.56E-06**	**4.67E-06**	2.09E-05
Gsta3	24.4048	23.3465	24.0418	22.8783	**2.28E-06**	0.016522	0.739313
Cyp2f2	21.0805	17.9324	21.5324	17.9836	**8.17E-08**	0.34302	0.077060
Hexim1	21.3395	20.9988	21.4487	20.7261	**6.79E-06**	0.32960	0.031957
Bach1	24.8354	24.5168	19.894	16.496	2.36E-05	**1.82E-12**	0.000143
Hk1; Hkdc1	23.523	23.309	23.095	22.461	0.000149	**1.47E-06**	0.026648
Adss	22.579	22.611	23.810	24.020	0.415842	**9.94E-08**	0.546994
Cd38	22.111	21.339	19.796	19.441	0.035242	**2.13E-07**	0.406045

Traf5	17.805	17.526	NaN*	NaN*	0.201162	**4.31E-07**	0.181736
Ralgapb	19.333	18.729	NaN*	NaN*	0.085544	**2.74E-13**	0.072106
Vcpkmt	19.074	18.624	19.873	19.858	0.0711526	**2.71E-07**	0.089757
Adi1	22.098	21.635	22.894	22.549	0.0044239	**3.00E-06**	0.633746
Tsfm	23.001	23.257	23.700	23.88	0.0033806	**1.50E-08**	0.550737
Arpin	23.519	23.252	22.805	22.015	0.0002949	**2.49E-07**	0.037623
Rab1b	23.704	23.222	22.383	22.154	0.0688147	**6.59E-06**	0.496483

* NaN: not a number.

Supplementary Table S7: Pathways significantly regulated by maternal diet, analyzed with reactome.org version 77.

**Pathway name**	**Entities ratio**	**Entities p-value**	**Entities FDR**	**Reactions ratio**
Glutathione conjugation	0.003	6.44E-7	1.74E-5	0.001
Biological oxidations	0.019	1.99E-6	2.58E-5	0.013
Phase II - Conjugation of compounds	0.01	1.71E-5	1.54E-4	0.005
CYP2E1 reactions	0.001	5.68E-3	2.47E-2	0
Fatty acids	0.001	7.73E-3	2.47E-2	0
Synthesis of Prostaglandins (PG) and Thromboxanes (TX)	0.001	7.73E-3	2.47E-2	0.002
Heme degradation	0.001	8.25E-3	2.47E-2	0.001
Xenobiotics	0.002	1.24E-2	2.98E-2	0.002
Metabolism	0.185	1.28E-2	2.98E-2	0.152
Metabolism of porphyrins	0.003	1.49E-2	2.98E-2	0.003
Arachidonic acid metabolism	0.005	3.01E-2	3.87E-2	0.004
Cytochrome P450 - arranged by substrate type	0.006	3.32E-2	3.87E-2	0.005
Oxygen-dependent proline hydroxylation of Hypoxia-inducible Factor Alpha	0.006	3.37E-2	3.87E-2	0.001
Cellular response to hypoxia	0.007	3.87E-2	3.87E-2	0.002
Regulation of PLK1 Activity at G2/M Transition	0.008	4.42E-2	4.42E-2	0.001
Activation of HOX genes during differentiation	0.008	4.62E-2	4.62E-2	0.003

Activation of anterior HOX genes in hindbrain development during early embryogenesis	0.008	4.62E-2	4.62E-2	0.003
Phase I - Functionalization of compounds	0.009	5.31E-2	5.31E-2	0.007
Fatty acid metabolism	0.015	8.91E-2	8.91E-2	0.014
G2/M Transition	0.017	9.81E-2	9.81E-2	0.006

Supplementary Table S8: Pathways significantly regulated by maternal genotype, analyzed with reactome.org version 77.

**Pathway name**	**Entities ratio**	**Entities p-value**	**Entities FDR**	**Reactions ratio**
Regulation of BACH1 activity	0.001	1.82E-4	1.64E-2	0.001
Defective HK1 causes hexokinase deficiency (HK deficiency)	0	3.3E-3	9.89E-2	0
Glycolysis	0.008	6.4E-3	9.96E-2	0.002
Glucose metabolism	0.01	1.02E-2	9.96E-2	0.004
Cytoprotection by HMOX1	0.011	1.28E-2	9.96E-2	0.003
Golgi Cisternae Pericentriolar Stack Reorganization	0.001	1.85E-2	9.96E-2	0
Protein methylation	0.001	2.07E-2	9.96E-2	0.001
Cellular response to chemical stress	0.014	2.15E-2	9.96E-2	0.005
Cytosolic iron-sulfur cluster assembly	0.002	2.5E-2	9.96E-2	0
Defective HDR through Homologous Recombination (HRR) due to PALB2 loss of function	0.002	2.61E-2	9.96E-2	0
Defective HDR through Homologous Recombination Repair (HRR) due to PALB2 loss of BRCA2/RAD51/RAD51C binding function	0.002	2.61E-2	9.96E-2	0
Defective HDR through Homologous Recombination Repair (HRR) due to PALB2 loss of BRCA1 binding function	0.002	2.61E-2	9.96E-2	0
Diseases of DNA Double-Strand Break Repair	0.002	2.61E-2	9.96E-2	0
Methionine salvage pathway	0.002	2.61E-2	9.96E-2	0.001
Resolution of D-loop Structures through Synthesis-Dependent Strand Annealing (SDSA)	0.002	3.25E-2	9.96E-2	0
Resolution of D-loop Structures through Holliday Junction Intermediates	0.002	3.89E-2	9.96E-2	0.001
Resolution of D-Loop Structures	0.003	3.99E-2	9.96E-2	0.001
Diseases of DNA repair	0.003	3.99E-2	9.96E-2	0.002

HDR through Single Strand Annealing (SSA)	0.003	4.21E-2	9.96E-2	0

Entities ratio: The total entities in the pathway divided by the total number of entities for the entire species for the selected molecular type.; Entities p-value: Probability that the overlap between the query and the pathway has occurred by chance.; Entities FDR: Probability corrected for multiple comparisons.; Reactions ratio: The total reactions in the pathway divided by the total number of the entire species for the selected molecular type.
